# Construction of Biocatalysts Using the P450 Scaffold for the Synthesis of Indigo from Indole

**DOI:** 10.3390/ijms24032395

**Published:** 2023-01-25

**Authors:** Yanqing Li, Yingwu Lin, Fang Wang, Jinghan Wang, Osami Shoji, Jiakun Xu

**Affiliations:** 1 College of Food Science and Technology, Shanghai Ocean University, Shanghai 201306, China; 2 Key Lab of Sustainable Development of Polar Fisheries, Ministry of Agriculture and Rural Affairs, Yellow Sea Fisheries Research Institute, Chinese Academy of Fishery Sciences, Lab for Marine Drugs and Byproducts of Pilot National Lab for Marine Science and Technology, Qingdao 266071, China; 3 School of Chemistry and Chemical Engineering, University of South China, Hengyang 421001, China; 4 College of Food Science and Engineering, Ocean University of China, Qingdao 266003, China; 5 Department of Chemistry, Graduate School of Science, Nagoya University, Furo-cho, Chikusa-ku, Nagoya 464-8602, Japan

**Keywords:** cytochromes P450BM3, dual-functional small molecule co-catalysis, hydroxylated indole, indigo, directed evolution

## Abstract

With the increasing demand for blue dyes, it is of vital importance to develop a green and efficient biocatalyst to produce indigo. This study constructed a hydrogen peroxide-dependent catalytic system for the direct conversion of indole to indigo using P450BM3 with the assistance of dual-functional small molecules (DFSM). The arrangements of amino acids at 78, 87, and 268 positions influenced the catalytic activity. F87G/T268V mutant gave the highest catalytic activity with *k_cat_* of 1402 min^−1^ and with a yield of 73%. F87A/T268V mutant was found to produce the indigo product with chemoselectivity as high as 80%. Moreover, F87G/T268A mutant was found to efficiently catalyze indole oxidation with higher activity (*k_cat_*/K_m_ = 1388 mM^−1^ min^−1^) than other enzymes, such as the NADPH-dependent P450BM3 (2.4-fold), the Ngb (32-fold) and the Mb (117-fold). Computer simulation results indicate that the arrangements of amino acid residues in the active site can significantly affect the catalytic activity of the protein. The DFSM-facilitated P450BM3 peroxygenase system provides an alternative, simple approach for a key step in the bioproduction of indigo.

## 1. Introduction

As one of the oldest dyes, indigo was initially extracted from a variety of plant species, such as Indigofera tinctoria, woad, and polygonum in India [1,2,3]. Due to the increasing demand for this blue dye, many efforts have been devoted to indigo production since the nineteenth century [4,5]. Adolph von Baeyer received the Nobel Prize in chemistry in 1905 for determining the molecular structure and synthetic routes of indigo [6]. Compared to general organic synthesis, biocatalysts, including a whole cell system, have offered an alternative for indigo production, and these environmentally friendly reactions can be carried out under mild conditions without the use of organic solvents, which have several advantages from the viewpoint of green chemistry [7,8,9,10,11,12,13,14,15,16,17,18,19,20,21,22,23,24]. Hemeproteins such as cytochrome P450s have been utilized as a biocatalyst and succeeded in directly producing indigo from indole [13,16,19,20,21,25,26]. However, these P450s generally require large quantities of expensive cofactors, NADPH or NADH, thus limiting their practical application [27]. Although NADPH-regeneration systems effectively reduce costs, the catalytic systems become complicated to apply to practical production. A whole-cell catalysis system using naphthalene dioxygenase (NDO) is one of the most practical. Still, this system is rather complicated because of the involvement of coenzymes to retard the side reaction [11,12].

**Figure 1:**
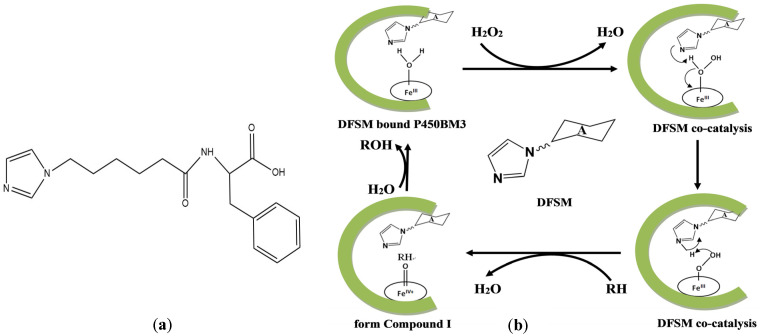
(**a**) The structure of the DFSM (Im-C6-Phe). (**b**) Proposed catalytic cycle of the artificial P450BM3 peroxygenase system, RH represents the substrate, and ROH represents the product.

P450BM3, a self-sufficient cytochrome P450 enzyme from *Bacillus megaterium* [28], can catalyze regional and stereoselective oxidation of inert C-H bonds in complex compounds under mild conditions, which has obvious advantages over chemical oxidation [29]. The cytochrome P450 catalytic cycle uses oxygen as an oxidant and the reducing coenzyme NAD(P)H provides two electrons [30,31]. The two-step sequential electron transfer results in a highly oxidized and reactive intermediate Compound I [32], which directly interacts with the substrate to produce substrate oxidation. Most cytochrome P450s follow the catalytic cycle, but they can also directly use hydrogen peroxide shunt pathway to complete the reaction, the intermediate can skip the electron transfer step and directly combine with hydrogen peroxide to form an intermediate. This class of P450 is also known as peroxidase [33]. Peroxidase not only use cheap hydrogen peroxide as oxidant, but also greatly simplify the catalytic cycle. However, wild type P450BM3 shows no catalytic activity for natural substrates with a chain length shorter than twelve carbons and cannot use hydrogen peroxide as the main oxidant [34]. Rational design and directed evolution strategies on P450 engineering enable it to catalyze a wider range of substrate hydroxylation [25,35,36,37]. Previously, an artificial P450BM3 peroxygenase system, which has a dual-functional small molecule (DFSM) installed in the active pocket, was developed to perform an H_2_O_2_-dependent monooxygenation of non-native substrates [38,39,40,41,42,43]. Compared with the native NADPH-dependent P450BM3, the DFSM-facilitated P450BM3 peroxygenase system simplifies the catalytic cycle of P450BM3 using green and inexpensive H_2_O_2_, making it more economical and practical [43,44]. Through this system, we have recently achieved hydroxylation of indole in the presence of DFSM (Im-C6-Phe) with H_2_O_2_ as an oxidant (Figure 1), opening up alternative avenues for synthetic indigo.

These results encouraged us to investigate the hydroxylation of indole by a series of P450BM3 mutants to produce indigo, while peroxidases themselves are not able to catalyze the hydroxylation of indole. To construct a simple and more economical reaction system for indigo production, we constructed a hydrogen peroxide-dependent catalytic oxidation system to directly convert indole to indigo using the P450BM3 scaffold.

## 2. Results and Discussion

### 2.1. Protein Expression and Characterization

P450BM3 mutants were expressed as a soluble holo-protein in *Escherichia coli* BL21 (DE3) and purified using the same procedure as the Wild-type P450BM3. The results of SDS-PAGE of P450BM3 and its mutants showed a single band around 55 kDa, consistent with the characteristics of P450BMP (Figure S1). Wild-type P450BM3 and its mutants formed a ferrous CO complex through the reduction of ferric heme upon the addition of Na_2_S_2_O_4_ and their UV-vis spectral changes under this condition (Figure S2) [45]. The concentrations of P450BM3 and its variants were measured by hemochrome binding assay [46].

### 2.2. Indole Oxidation Catalyzed by the P450BM3 Peroxygenase System

**Figure 2:**
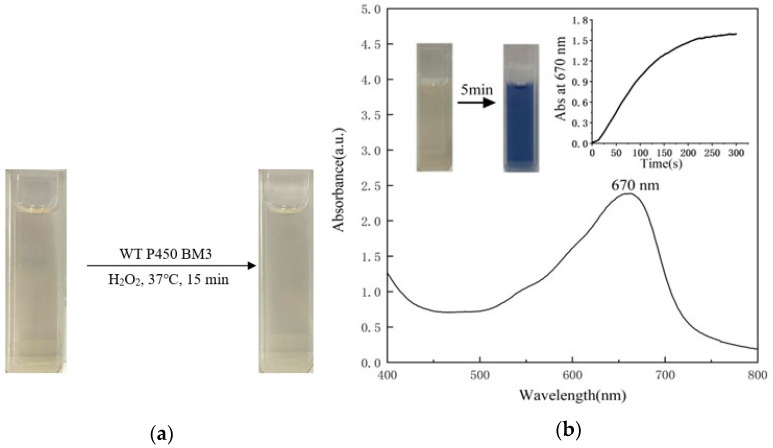
(**a**) The oxidation of indole (1 mM) catalyzed by WT P450 BM3 (0.5 μM) with H_2_O_2_ (30 mM) as an oxidant; (**b**) UV-vis spectral changes upon the coupling oxidation of indole catalyzed by F87A P450BM3 mutant. Time-dependent changes of the absorbance at 670 nm and the color change of the solution are shown as insets.

**Figure 3:**
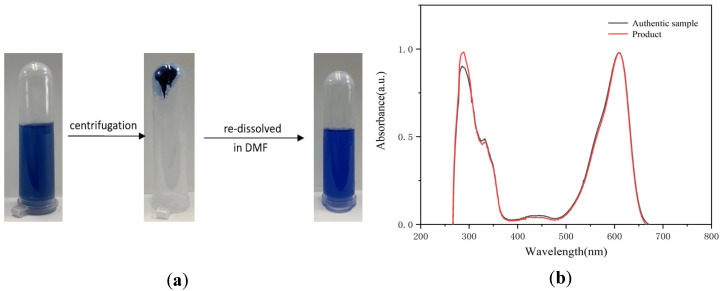
(**a**) After centrifuging the reaction solution of indole, as catalyzed by F87A P450BM3 mutant, the resulting precipitate was redissolved in DMF to obtain a bright blue solution; (**b**) UV-Vis spectra of the authentic indigo sample (black) and the biosynthesized indigo (red) in dimethylformamide (DMF).

**Figure 4:**
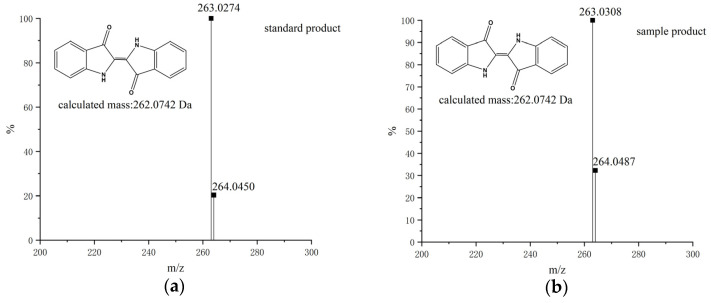
UPLC-MS spectra of the oxidation product of standard product (**a**) and indole in precipitation catalyzed by F87G/T268V P450BM3 mutant (**b**). Indigo (C_16_H_10_N_2_O_2_), Calculated: 262.0742 Da; Observed: 263.0308 Da ([M + H]^+^).

The optimal reaction conditions were selected with 37 °C, pH 8.0, and a hydrogen peroxide concentration of 30 mM. Initially, we investigated the oxidation of indole by wild-type P450BM3 in the presence of DFSM and found that the wild-type had no catalytic ability (Figure 2a). Then we catalyzed the oxidation of indole with the P450BM3 mutant F87A mutant. When P450BM3 mutants oxidized indole, the color of the reaction mixture turned blue (Figure 2b). As monitored by UV-vis spectroscopy, a clear increase at around 670 nm was observed upon the addition of hydrogen peroxide. By centrifugation of the blue solution, a precipitate was generated, well-dissolved in DMF, displaying a bright blue color (Figure 3a). The UV-vis spectrum of the isolated blue product dissolved in DMF was identical to that of indigo, with an absorption maximum of 610 nm (Figure 3b). The HPLC analysis of the extract of the reaction mixture monitored at 280 nm gave a peak generated after the reaction at 7.91min might be assignable to indigo. The blue band silica was collected from the plate for the mass spectrometry detection, and the mass spectrum gave a peak at m/z 263.0308 [M + H]^+^ (Figure 4) that agrees well with indigo (calculated mass for C_6_H_10_N_2_O_2_ 262.0742 Da). ^1^H NMR (DMSO-*d*_6_, 600 MHz) results were δ6.98 (t, 2H), 7.35 (d, 2H), 7.53 (t, 2H), 7.63 (d, 2H), 10.50 (s, 2H). The ^1^H NMR spectra of the product also agree well with the literature [47]. These results indicated the formation of indigo from indole.

### 2.3. Kinetic Study on the Indigo Formation and the Site-Directed Mutagenesis

**Figure 5:**
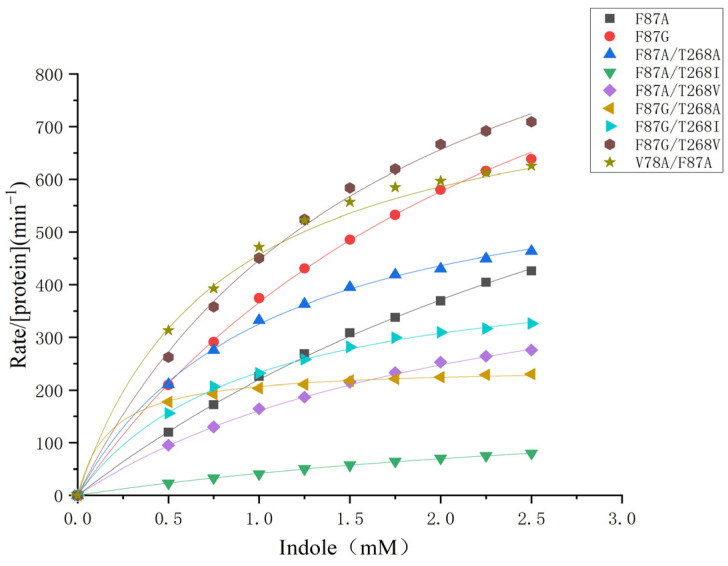
The plots of catalytic reaction rate of the indole formation by a series of P450BM3 mutants against the concentration of indole.

The catalytic properties of mutants were evaluated by Michaelis–Menten kinetics. Wild-type P450BM3 showed no catalytic ability against indole oxidation, which could be attributed to the existence of phenylalanine 87 in the substrate binding pocket preventing the access of foreign substrate. As shown in Figure 5, the k_obs_ values and the concentrations of indole were plotted and fitted to the Michaelis–Menten equation. To improve the catalytic ability, we first replaced the aromatic ring of phenylalanine 87 with smaller alanine and glycine. The resulting F87A and F87G mutants created a wider hydrophobic binding space in the heme pocket of P450BM3 for the access of indole. In Table 1, kinetic parameters (K_m_, *k_cat_*, and *k_cat_*/K_m_) of F87A and other nine P450BM3 mutants are summarized and compared with the literature, which includes the kinetic parameters of A74G/F78V/L188Q D168N/A255V/K440N and other six P450BM3 mutants, myoglobin and neuroglobin mutantsF87A, and F87G mutants showed remarkably improved catalytic activity towards the reaction with *k_cat_* of 1007 min^−1^ and 1304 min^−1^. The catalytic efficiency of F87A and F87G mutants was 296 mM^−1^ min^−1^ and 466 mM^−1^ min^−1^, respectively. To further improve the catalytic ability of the F87A and F87G mutants, we replaced Val78 with smaller side chain alanine and constructed a hydrophobic space inside the heme pocket of P450BM3 by replacement Thr268 with alanine, isoleucine, and valine. The catalytic activity of the V78A/F87A mutant (1179 min^−1^) is 17% higher than that of the F87A mutant (1007 min^−1^), indicating the reduction of the amino acid size at position 78 of the F87A mutant is beneficial to the reaction. However, V78A/F87G mutant showed dramatically decreased catalytic ability. It can be seen the replacement of Val78 and Thr268 with hydrophobic groups such as alanine(A), isoleucine(I), and Valine(V) facilitates the binding of indole to the heme active site during the reaction. The K_m_ of most of the double mutants was smaller than that of the parent enzyme. The catalytic efficiency of the F87G/T268A mutant was 1388 mM^−1^ min^−1^, which is 3-fold higher than that of F87G. The introduction of alanine at position 268 of the F87G mutant contributed to the binding of indole to the protein with a much smaller K_m_ 0.17 mM, resulting in improved catalytic efficiency. F87G/T268A mutant has thus been the most efficient biocatalyst so far with respect to NADPH-dependent P450BM3 enzymes and hydrogen peroxide-dependent oxidation, such as myoglobin and neuroglobin, which is 2.4-fold than the most effective NADPH-dependent P450BM3 mutant A74G/F87V/L188Q/E435T (*k_cat_*/K_m_ = 577 mM^−1^ min^−1^), 117-fold than that of the Mb double mutant F43Y/H64D (*k_cat_*/K_m_ = 11.86 mM^−1^ min^−1^) and 32-fold than that of the Ngb triple mutant A15C/H64D/F49Y (*k_cat_*/K_m_ = 43.25 mM^−1^ min^−1^) [23,24,48].

### 2.4. Analysis of the Mixture after the Oxidation Reaction

**Figure 6:**
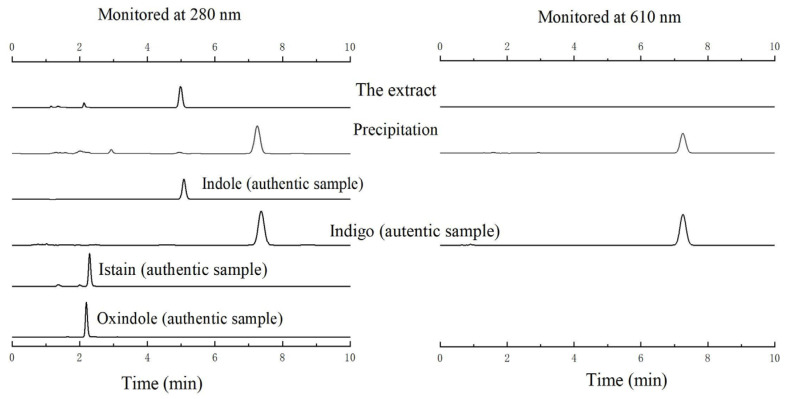
HPLC analysis of the extract of the reaction mixture catalyzed by the F87A P450BM3 mutant monitored at 280 nm (**left**) and 610 nm (**right**). HPLC traces of authentic indole, indigo, isatin, and oxindole samples were shown for comparison.

**Scheme 1:**
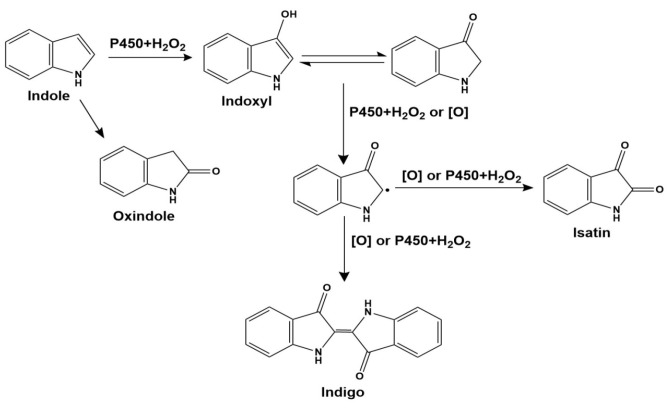
A plausible reaction mechanism for indigo formation from indole catalyzed by P450BM3 mutants.

Indigo was the product of oxidative dimerization of two 3-hydroxyindole molecules. To determine the regioselectivity of each mutant, 0.5 μM purified P450BM3 mutants were applied to hydroxylate indole and produced a sufficient amount of the hydroxylated product. The HPLC analysis of the extract of the reaction mixture monitored at 280 nm and 610 nm gave a peak at 7.91 min corresponding to indigo. In contrast, a couple of peaks assignable to isatin (2.29 min) and oxindole (2.19 min) were also observed (Figure 6 and Figure S3). Oxidation of indole-formed side products such as isatin and oxindole. These side products were likely formed with a mechanism similar to that proposed for Mb by Watanabe and co-workers [17]. Although the side product formation was not suppressed, it is noteworthy that indigo can be easily isolated from the reaction mixture by centrifugation or filtration because indigo is readily isolated as precipitate by allowing the reaction mixture to stand at room temperature for several hours. The indigo separated by centrifugation showed no appreciable amount of side products in the HPLC measurement, indicating that indigo can be easily purified without using column chromatography in our reaction system. A plausible reaction mechanism for the formation of indigo from indole catalyzed by the P450BM3 mutant is shown in Scheme 1. The formation of indigo beings with the hydroxylation of indole at its 3-position, followed by one-electron-oxidation reactions of indoxyl and its radical coupling reaction. The binding structure of indole with P450BM3 and its mutants significantly affected the amount of indigo produced by indole hydroxylation. When the indole was located above the heme, and its C-3 carbon was closest to heme iron, the yield of indigo was higher.

HPLC examined the number of products and the chemoselectivity of P450BM3 mutants at 280nm and 610 nm (Table 2). Single mutants F87A and F87G can catalyze the hydroxylation of indole to indigo with yields of 37% and 58%, respectively. Then we check the influence of amino acid size at position 78 on chemoselectivity by mutating Val78 to smaller alanine. The yield of V78A/F87A (58%) is 1.6 times higher than that of F87A (37%), and we assumed that the increased space at position 78 contributes to the binding of the foreign substrate, thereby increasing the yield. We also prepared the double mutants F87A/T268A, F87A/T268I, F87A/T268V, F87G/T268A, F87G/T268I, and F87G/T268V mutant by mutation of Thr268 to alanine, isoleucine and valine based on mutants F87A and F87G. The yields of double mutants F87A/T268V (60%) and F87G/T268V (73%) were 1.6-fold and 1.25-fold higher than F87A and F87G, whereas other double mutants F87A/T268A (17%), F87A/T268I (14%), F87G/T268A (8%), F87G/T268I (31%) showed lower yields than the parental single mutants F87A and F87G.

Regarding chemoselectivity, mutant F87A and F87G catalyzed the hydroxylation of indole to indigo with 49% and 62% chemoselectivity, respectively. The chemoselectivity of double mutant F87A/T268V (80%), F87G/T268I (78%), and F87G/T268V (75%) were increased by 1.6-fold, 1.25-fold, and 1.2-fold compared with the single parent mutant F87A and F87G. The chemoselectivity of three double mutants, F87A/T268A, F87A/T268I, and F87G/T268A, was 46%, 42%, and 44%, respectively, indicating that the reduction of the amino acid size at position 268 and the increase of the hydrophobicity did not effectively improve the chemoselectivity. When Val78 was mutated to a smaller amino acid amine, the chemoselectivity of the V78A/F87A mutant was improved to 59%. This suggests that the hydrophobic cavity facilitates the reaction of the foreign substrate in the active site. At the same time, the size of the cavity also affects the binding of the substrate to the active site.

### 2.5. Molecular Modeling Simulation

**Figure 7:**
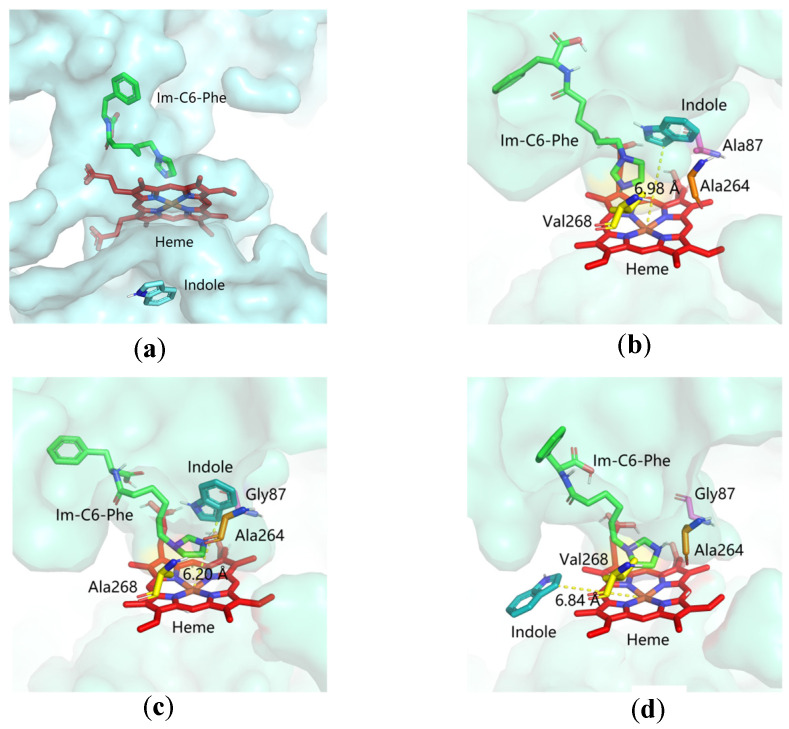
The docking simulation of indole to the active site of (**a**) WT, (**b**) F87A/T268V, (**c**) F87G/T268A, and (**d**) F87G/T268V P450BM3 mutants. Indole (cyan), heme (red), and DFSM (green) are represented as stick models. The amino acid residues at positions 87, 264 positions and 268 positions are shown in purple, orange and yellow, respectively.

To investigate the structural interaction between the protein and substrate, we performed molecular docking studies of indole binding to wild-type P450BM3 and double mutants, F87A/T268V, F87G/T268A, and F87G/T268V mutants. The simulated structures of P450BM3 mutants were used as rigid receptors for docking of indole in the Autodock 4 program. The calculated binding energies for each substrate are shown in Tables S1–S3. Out of the ten simulated complexes, the ones with the lowest energy of each mutant are shown in Figure 7b,d. Figure 7a shows that when the wild-type P450BM3 combined with indole, the indole molecule could not reach the top of the heme of the wild-type P450BM3, indicating that the wild-type P450BM3 does not have catalytic activity. The mutation at 87 and 268 positions of the P450BM3 could influence both the location and orientation of indole in the active site in the presence of the DFSM molecule. The structure revealed that the distal Ala264 adopted an open conformation upon substrate binding in these three mutants. The indole molecule was bound to the heme distal pocket without alteration of the heme structure. We observed a clear difference in the location and the orientation of indole in these three P450BM3 mutants. Indole was accommodated deep inside the heme pocket, and the benzene ring of indole was positioned inside the heme cavity of F87A/T268V and F87G/T268A mutants. In contrast, the pyrrole ring was located toward the center of the heme of the F87G/T268V mutant. The distance between the C-3 carbon of indole and the heme iron of the F87A/T268V, F87G/T268A, and F87G/T268V mutants were 6.98 Å, 6.20 Å and 6.84 Å, respectively. The mutation at 87 and 268 positions of the P450 could influence both the location and orientation of the in-dole in the active site in the presence of the DFSM molecule. In the comparison mutants, the difference in the distance, position, and orientation between the C-3 carbon of in-dole and heme iron could affect the substrate binding and electron transfer of indole, thus improving the fixation of indole and thus improving catalytic activity. A similar phenomenon was observed in other literature [53,54].

## 3. Materials and Methods

### 3.1. Chemicals

Indigo was obtained from Macklin (Shanghai, China), and oxindole was purchased from Energy Chemical Industries (Shanghai, China). Indole, isatin, and indigo derivatives were obtained from Aladdin (Shanghai, China). All chemicals purchased from commercial sources were used without further purification.

### 3.2. Instrument

UV-vis spectra were recorded on a UV-2550 diode array spectrometer (Shimadzu, Tokyo, Japan). High-performance liquid chromatography (HPLC) analysis was carried out using a LC-20A equipped with an SPD-M20A UV-visible detector (Shimadzu, Tokyo, Japan). Ultra-performance liquid chromatography (UPLC) -MS was analyzed in the LC-20AD/AB SCIEX QTRAP 5500 system (Shimadzu, Tokyo, Japan), using a reverse-phase C18 column (CAPCELL PAK C18 MGII 5.0 µm, 2.0 mm × 150 mm).

### 3.3. Site-Directed Mutagenesis, Cultivation, and Purification of P450BM3 Mutants

All mutations were made by PCR-based site-directed mutagenesis and verified by DNA sequencing. Three sites around the substrate binding channel were selected for mutation by P450BM3 modeling. V78 and F87 are located at the substrate channel close to the active center and T268 in the I helix knot region [40,44,45,46,47,48,49,50,51,52,53,54,55,56]. Primers for each site are listed in Table 3. The pET-28a (+) vectors containing BMP and its variants were expressed in *Escherichia coli* BL21 (DE3) cells.

The cells were cultivated in an LB medium containing 50 µg/mL kanamycin. The cultures were grown at 37 °C with 200 rpm to the OD600 and reached 0.8~1.0. The expression was induced for 16–20 h at 30 °C by the addition of IPTG (1 mM), δ-aminolevulinic acid hydrochloride (0.5 mM), and FeCl_3_ (0.5 mM). The cultures were centrifuged at 6000 rpm for 10 min, and the cell pellets were resuspended in buffer A (100 mM KPi, 500 mM NaCl, pH 7.4) and lysed by sonication. Cell debris was removed by centrifugation at 10,000× *g* for 60 min. Purification was done by Ni-NTA metal-affinity chromatography. The crude cell extraction was applied to a 5 mL bed volume column pre-equilibrated with 4% buffer A and 96% buffer B (100 mM KPi, 500 mM NaCl, 500 mM imidazole, pH 7.4). Nonspecifically bound proteins were washed from the column with 5 column volumes of 6% buffer A and 94% buffer B, and the bound protein was eluted with 60% buffer A and 40% buffer B. The purified protein solution was dialyzed with buffer C (100 mM KPi, 100 mM NaCl, pH 7.4). The purity of BMP and its variants was checked by SDS-PAGE (Figure S1).

### 3.4. Catalytic Activity Assay

The reaction was carried out in 50 mM potassium phosphate buffer (pH 8.0) containing 0.5–2.5 mM indole and 0.5 μM protein and 0.5 mM DFSM (dissolved in 50 mM pH 8.0 phosphate buffer) with a final volume of 1 mL. The mixture was pre-incubated at 37 °C for 5 min, and the reaction was initiated by the addition of hydrogen peroxide (30 mM). The absorption monitored the reaction at 670 nm (ε = 4.8 mM^−1^ cm^−1^) on a Shimadzu UV-2550 diode array spectrometer. The initial rates as a function of indole concentrations were fitted to the Michaelis–Menten equation: v/[protein] = *k_cat_*[substrate]/(K_m_ + [substrate]) to obtain the kinetic parameters *k_cat_* and K_m_. A control study of wild-type P450BM3 was performed under the same conditions.

### 3.5. Analysis of the Reaction Mixture after the Oxidation

We optimized the reaction conditions by changing the temperature (25–45 °C), pH (6.0–10.0), and concentration of hydrogen peroxide (5 mM−35 mM). After a 10 min reaction catalyzed by P450BM3 mutants with a final volume of 1 mL, the reaction mixture was centrifuged for 20 min (10,000 r min^−1^), producing a blue precipitate and light-yellow supernatant. The blue precipitate was redissolved in DMF, and the aliquots were injected into a Waters Sunfire^TM^ C18 HPLC column (4.6 × 250 mm). The column was eluted isocratically with acetonitrile and 20 mM potassium phosphate (pH 7.0) (50:50, *v/v*) at a flow rate of 1 mL/min at 30 °C. The eluent was monitored at 280 nm and 610 nm, and the peaks corresponding to indole, indigo, isatin, and oxindole were assigned using authentic samples. The yield of indigo was calculated as follows: ([Indigo] × 2/[The amount of indole added to the system]) × 100%. Moreover, the chemoselectivity of indigo formation was also estimated as follows: ([Indigo] × 2/[Consumed indole]) × 100%.

### 3.6. Isolation and Characterization of Indigo

To a solution of 0.5 mM P450BM3 mutants in 1mL of 50 mM potassium phosphate buffer, (pH 8) containing 1 mM indole was added 30 mM hydrogen peroxide to start the reaction. The reaction mixture was incubated at 37 °C for 10 min. The resulting mixture was subjected to a silica gel plate chromatography (1 mm × 20 cm × 20 cm) and developed with CHCl_3_/CH_3_OH (50:1, *v/v*). A blue band of silica was collected from the plate and extracted with CHCl_3_/CH_3_OH (1:1, *v/v*). After filtration, the filtrate was redissolved in chloroform or acetonitrile and measured by UPLC-MS, UV-vis spectroscopy, and ^1^H NMR Spectroscopy.

### 3.7. Molecular Modeling

The structures of F87A/T268V, F87G/T268A, and F87G/T268V mutants were calculated based on the crystal structure of the F87A/T268A mutant (PDB code: 3DGI) by Insight II 2000/Discover 3 with Extensible Systematic Force Field (ESFF). The simulated structures of P450BM3 mutants were used for docking indole in the Autodock 4 [57]. The P450BM3 mutants were first docked with dual-functional small molecule Im-C6-Phe, and the optimal structure was then docked with the substrate indole. Amino acid residues Phe87, Ala264, Thr268, and Ala328 were set as flexible residues, and the heme iron was set in the center. The structure of dual-functional small molecule and indole were generated by ChemDraw. Docked conformations were ranked automatically by Autodock 4 using a free-energy scoring function. The docking results were then visualized using PYMOL [58].

## 4. Conclusions

In summary, we have successfully transformed indole into indigo by employing a DFSM-facilitated P450BM3 peroxygenase system using hydrogen peroxide as an oxidant. The arrangements of amino acid residues in the active site can significantly affect the catalytic activity of the protein. The amino acid residues at 78, 87, and 268 positions in the active site are crucial for catalytic efficiency. F87G/T268V mutant gave the highest *k_cat_* (1402 min^−1^), and with a yield as high as 73%, F87G/T268A mutant gave the highest *k_cat_*/K_m_ (1388 mM^−1^ min^−1^) and with the chemoselectivity up to 80%. The established convenient and green method could serve as the biocatalyst for indigo production.

## Figures and Tables

**Table 1:** Kinetic parameters of P450BM3 and its mutants, Mb and Ngb, catalyzed indole oxidation.

Enzymes	DFSM	*k_cat_* (min^−1^)	K_m_ (mM)	*k_cat_*/K_m_ (mM^−1^min^−1^)
WT P450BM3	Im-C6-Phe ^a^	nd	nd	nd
F87A P450BM3	Im-C6-Phe	1007 ± 39.96	3.4 ± 0.20	296
F87G P450BM3	Im-C6-Phe	1304 ± 34.09	2.8 ± 0.11	466
V78A/F87A P450BM3	Im-C6-Phe	1179 ± 18.66	1.59 ± 0.05	742
V78A/F87G P450BM3	Im-C6-Phe	nd	nd	nd
F87A/T268A P450BM3	Im-C6-Phe	687 ± 10.43	1.1 ± 0.04	625
F87A/T268I P450BM3	Im-C6-Phe	223 ± 6.80	4.4 ± 0.18	50.68
F87A/T268V P450BM3	Im-C6-Phe	536 ± 13.81	2.33 ± 0.10	230
F87G/T268A P450BM3	Im-C6-Phe	236 ± 1.47	0.17 ± 0.01	1388
F87G/T268I P450BM3	Im-C6-Phe	478 ± 6.62	1.07 ± 0.03	447
F87G/T268V P450BM3	Im-C6-Phe	1402 ± 44.90	2.1 ± 0.12	668
A74G/F78V/L188Q/D168N/A255V/K440N P450BM3 [49]	-	187	1.5	124
A74G/ F87V/L188Q/D168L [50]	-	387	0.90	420
A74G/ F87V/L188Q/D168H [50]	-	600	1.20	500
A74G/ F87V/L188Q/E435T [50]	-	450	0.78	577
A74G/F87V/L188Q/E435T/D168R P450BM3 [48]	-	189	0.68	278
A74G/ F87V/L188Q/D168L/E435T P450BM3 [51]	-	28.15	1.72	16.37
D168L/E435T/V445A P450BM3 [52]	-	73.81	2.99	24.77
H64D/V68I/I107V Mb ^b^ [17]	-	71.9	11.2	6.42
F43Y/H64D Mb [23]	-	5.22	0.44	11.86
A51C/H64D/F49Y Ngb ^c^ [24]	-	6.92	0.16	43.25

^a^ Im-C6-Phe: N-(ω-imidazol-1-yl hexanoyl)-L-phenylalanine. ^b^ Mb: myoglobin. ^c^ Ngb: neuroglobin.

**Table 2:** The amount of indigo and indole after 10 min reaction and the chemoselectivity to indigo estimated by HPLC by P450BM3 mutants.

Enzymes	Indigo (μM)	Indole (μM)	Indigo Yield (%)	Chemoselectivity (%)
F87A	185	241	37 ± 1.62	49 ± 1.92
F87A/T268A	83	635	17 ± 1.85	46 ± 2.26
F87A/T268I	69	672	14 ± 0.94	42 ± 1.48
F87A/T268V	299	252	60 ± 2.77	80 ± 3.62
F87G	292	50	58 ± 2.58	62 ± 2.67
F87G/T268A	41	811	8 ± 0.95	44 ± 1.78
F87G/T268I	156	543	31 ± 0.10	78 ± 2.20
F87G/T268V	365	26	73 ± 1.47	75 ± 1.69
V78A/F87A	289	24	58 ± 3.10	59 ± 1.31

**Table 3:** Primers used in mutagenesis.

Mutation	Sequence (5′-3′)
F87A-F	GCGACAAGCTGGACGCATGAAAAAAATTG
F87G-F	GGTACAAGCTGGACGCATGAAAAAAATTGGAA
F87-R	TAACCCGTCTCCTGCAAAATCACGTACAA
T268A-F	GCGACAAGTGGTCTTTTATCATTTGC
T268V-F	GTGACAAGTGGTCTTTTATCATTTGC
T268I-F	ATCACAAGTGGTCTTTTATCATTTGC
T268-R	TTCGTGTCCCGCAATTAAGAATG
V78A-F	GCACGTGATTTTGCAG
V78-R	CGCTTGACTTAAGTTTTTATC

## Data Availability

Not applicable.

## Supplementary Materials

The following supporting information can be downloaded at: https://www.mdpi.com/article/10.3390/ijms24032395/s1.

## References

[1] Taylor G.W. (1986). Natural dyes in textile applications. Color. Technol..

[2] Ferreira E.S.B., Hulme A.N., McNab H., Quye A. (2004). The natural constituents of historical textile dyes. Chem. Soc. Rev..

[3] Gilbert K.G., Maule H.G., Rudolph B., Lewis M., Vandenburg H., Sales E., Tozzi S., Cooke D.T. (2010). Quantitative analysis of indigo and indigo precursors in leaves of *Isatis* spp. and *Polygonum tinctorium*. Biotechnol. Prog..

[4] Cotson S., Holt S.J. (1958). Studies in enzyme cytochemistry. IV. kinetics of aerial oxidation of indoxyl and some of its halogen derivatives. Proc. R. Soc. Biol. Sci..

[5] Ziegler E., Kappe T. (1964). A new synthesis of indigo. Angew. Chem. (Int. Ed.).

[6] Baeyer A.D.A., Drewsen V.B.a. (1882). Darstellung von Indigblau aus Orthonitrobenzaldehyd. Ber. Der Dtsch. Chem. Ges..

[7] Ensley B.D., Gibson D.T., Laborde A.L. (1982). Oxidation of naphthalene by a multicomponent enzyme system from Pseudomonas sp. strain NCIB 9816. J. Bacteriol..

[8] Ensley B., Ratzkin B., Osslund T., Simon M., Wackett L. (1983). Expression of naphthalene oxidation genes in *Escherichia coli* results in the biosynthesis of indigo. Science.

[9] Murdock D., Ensley B.D., Serdar C., Thalen M. (1993). Construction of metabolic operons catalyzing the De Novo biosynthesis of indigo in *Escherichia coli*. Biotechnology.

[10] Bialy H. (1997). Biotechnology, bioremediation, and blue genes. Nat. Biotechnol..

[11] Gillam E.M. (1999). Formation of indigo by recombinant mammalian cytochrome P450. Biochem. Biophys. Res. Commun..

[12] Gillam E.M.J., Notley L.M., Cai H., De Voss J.J., Guengerich F.P. (2000). Oxidation of indole by cytochrome P450 enzymes. Biochemistry.

[13] Li Q.S., Schwaneberg U., Fischer P., Schmid R.D. (2015). Directed evolution of the fatty-acid hydroxylase P450 BM-3 into an indole-hydroxylating catalyst. Chemistry.

[14] Nakamura K., Martin M.V., Guengerich F.P. (2001). Random mutagenesis of human cytochrome p450 2A6 and screening with indole oxidation products. Arch. Biochem. Biophys..

[15] Li H.M., Mei L.H., Urlacher V.B., Schmid R.D.l. (2008). Cytochrome P450 BM-3 evolved by random and saturation mutagenesis as an effective indole-hydroxylating catalyst. Appl. Biochem. Biotechnol..

[16] Wolk J.L., Frimer A.A. (2010). Preparation of tyrian purple (6,6′-Dibromoindigo): Past and present. Molecules.

[17] Xu J.K., Shoji O., Fujishiro T., Ohki T., Ueno T., Watanabe Y. (2012). Construction of biocatalysts using the myoglobin scaffold for the synthesis of indigo from indole. Catal. Sci. Echnol..

[18] Nagayama H., Sugawara T., Endo R., Ono A., Kato H., Ohtsubo Y., Nagata Y., Tsuda M. (2015). Isolation of oxygenase genes for indigo-forming activity from an artificially polluted soil metagenome by functional screening using Pseudomonas putida strains as hosts. Appl. Microbiol. Biotechnol..

[19] Brixius-Anderko S., Hannemann F., Ringle M., Khatri Y., Bernhardt R. (2017). An indole-deficient *Escherichia coli* strain improves screening of cytochromes P450 for biotechnological applications. Biotechnol. Appl. Biochem..

[20] Fiorentini F., Hatzl A.M., Schmidt S., Savino S., Glieder A., Mattevi A. (2018). The extreme structural plasticity in the CYP153 subfamily of P450s directs development of designer hydroxylases. Biochemistry.

[21] Kim J., Lee P.G., Jung E.O., Kim B.G. (2018). In vitro characterization of CYP102G4 from Streptomyces cattleya: A self-sufficient P450 naturally producing indigo. Biochim. Biophys. Acta Proteins Proteom..

[22] Fabara A.N., Fraaije M.W. (2020). An overview of microbial indigo-forming enzymes. Appl. Microbiol. Biotechnol..

[23] Liu C., Xu J.K., Gao S.Q., He B., Wei C.W., Wang X.J., Wang Z.H., Lin Y.W. (2018). Green and efficient biosynthesis of indigo from indole by engineered myoglobins. RSC Adv..

[24] Chen L., Xu J.K., Li L.Z., Gao S.Q., Wen G.B., Lin Y.W. (2022). Design and engineering of neuroglobin to catalyze the synthesis of indigo and derivatives for textile dyeing. Mol. Syst. Des. Eng..

[25] Oliver C.F., Modi S., Sutcliffe M.J., Primrose W.U., Lian L.Y., Roberts G.C.K. (1997). A single mutation in cytochrome P450 BM3 changes substrate orientation in a catalytic intermediate and the regiospecificity of hydroxylation. Biochemistry.

[26] Li Q.S., Schwaneberg U., Fischer M., Schmitt J., Schmid R.D. (2001). Rational evolution of a medium chain-specific cytochrome P-450 BM-3 variant. Biochim. Biophys. Acta (BBA) Protein Struct. Mol. Enzymol..

[27] Wichmann R., Vasic-Racki D. (2005). Cofactor regeneration at the lab scale. Technol. Transf. Biotechnol..

[28] Whitehouse C.J.C., Bell S.G., Wong L.-L. (2012). P450(BM3) (CYP102A1): Connecting the dots. Chem. Soc. Rev..

[29] Green M.T. (2009). C-H bond activation in heme proteins: The role of thiolate ligation in cytochrome P450. Curr. Opin. Chem. Biol..

[30] Meunier B., Visser S.P.d., Shaik S. (2004). Mechanism of oxidation reactions catalyzed by cytochrome P450 enzymes. Chem. Rev..

[31] Joyce M.G., Ekanem I.S., Roitel O., Dunford A.J., Neeli R., Girvan H.M., Baker G.J., Curtis R.A., Munro A.W., Leys D. (2012). The crystal structure of the FAD/NADPH-binding domain of flavocytochrome P450 BM3. FEBS J..

[32] Modi A.R., Dawson J.H. (2015). Oxidizing intermediates in P450 catalysis: A case for multiple oxidants. Adv. Exp. Med. Biol..

[33] Munro A.W., McLean K.J., Grant J.L., Makris T.M. (2018). Structure and function of the cytochrome P450 peroxygenase enzymes. Biochem. Soc. Trans..

[34] Miura Y., Fulco A.J. (1975). Omega-1, Omega-2 and Omega-3 hydroxylation of long-chain fatty acids, amides and alcohols by a soluble enzyme system from Bacillus megaterium. Biochim. Biophys. Acta.

[35] Joyce M.G., Girvan H.M., Munro A.W., Leys D. (2004). A single mutation in cytochrome P450 BM3 induces the conformational rearrangement seen upon substrate binding in the wild-type enzyme. J. Biol. Chem..

[36] Li Q.S., Ogawa J., Schmid R.D., Shimizu S. (2005). Indole hydroxylation by bacterial cytochrome P450 BM-3 and modulation of activity by cumene hydroperoxide. Biosci. Biotechnol. Biochem..

[37] Di S.Y., Fan S.X., Jiang F.J., Cong Z.Q. (2022). A unique P450 peroxygenase system facilitated by a dual-functional small molecule: Concept, application, and perspective. Antioxidants.

[38] Shoji O., Watanabe Y. (2014). Peroxygenase reactions catalyzed by cytochromes P450. J. Biol. Inorg. Chem..

[39] Ma N.N., Chen Z.F., Chen J., Chen J.F., Cong Z. (2018). Dual-functional small molecules for generating an efficient cytochrome P450BM3 peroxygenase. Angew. Chem. Int. Ed..

[40] Chen J., Kong F.H., Ma N.N., Zhao P.X., Liu C., Wang X.L., Cong Z.Q. (2019). Peroxide-driven hydroxylation of small alkanes catalyzed by an artificial P450BM3 peroxygenase system. ACS Catal..

[41] Xu J.K., Wang C.L., Cong Z.Q. (2019). Strategies for substrate-regulated P450 catalysis: From substrate engineering to co-catalysis. Chemistry.

[42] Willot S.J.P., Tieves F., Girhard M., Urlacher V.B., Hollmann F., de Gonzalo G. (2019). P450BM3-catalyzed oxidations employing dual functional small molecules. Catalysts.

[43] Zhang X.D., Peng Y.Q., Zhao J., Li Q., Li A.T. (2020). Bacterial cytochrome P450-catalyzed regio- and stereoselective steroid hydroxylation enabled by directed evolution and rational design. Bioresour. Bioprocess..

[44] Acevedo-Rocha C.G., Gamble C., Lonsdale R., Li A., Nett N., Hoebenreich S., Lingnau J.B., Wirtz C., Farès C., Hinrichs H. (2018). P450-catalyzed regio- and diastereoselective steroid hydroxylation: Efficient directed evolution enabled by mutability landscaping. ACS Catal..

[45] Omura T., Sato R. (1964). The carbon monoxide-binding pigment of liver microsomes. II. solubilization, purification, and properties. J. Biol. Chem..

[46] Wang Z.J., Renata H., Peck N.E., Farwell C.C., Coelho P.S., Arnold F.H. (2014). Improved cyclopropanation activity of histidine-ligated cytochrome P450 enables the enantioselective formal synthesis of levomilnacipran. Angew. Chem. Int. Ed..

[47] Guengerich F.P., Sorrells J.L., Schmitt S., Krauser J.A., Aryal P., Meijer L. (2004). Generation of new protein kinase inhibitors utilizing Cytochrome P450 mutant enzymes for indigoid synthesis. J. Med. Chem..

[48] Hu S., Yu Q., Mei L.H., Yao S.J., Jin Z.H. (2009). Semi-rational directed evolution in improving indole-hydroxylation ability of cytochrome P450 BM-3. CIESC J..

[49] Li H.M., Mei L.H., Urlacher V., Schmid R. (2005). Cytochrome P450BM-3 mutants with improved catalytic properties of hydroxylating indole to indigo by error-prone PCR. Prog. Biochem. Biophys..

[50] Li H.M., Mei L.H., Gao S.Y. (2008). Amino acid mutation effect on indole-hydroxylating ability of cytochrome P450 BM-3. J. Chem. Eng. Chin. Univ..

[51] Zhang P.P., Hu S., Mei L.H., Lei Y.L., Jin Z.H., Yao S.J. (2013). Improving indole-hydroxylation ability of cytochrome P450 BM-3 by site-directed mutagenesis. CIESC J..

[52] Zhang P.P., Hu S., Mei L.H., Lei Y.L., Jin Z.H., Yao S.J. (2014). A triple mutant improving activity of cytochrome P450 BM-3 to catalye indole hydroxylation: D168L/E435T/V445A. CIESC J..

[53] Reinhard F.G.C., Lin Y.-T., Stańczak A., Visser S.P.d. (2020). Bioengineering of cytochrome P450 OleTJE: How does substrate positioning affect the product distributions?. Molecules.

[54] Lin Y.T., de Visser S.P. (2021). Product distributions of cytochrome P450 OleTJE with phenyl-substituted fatty acids: A computational study. Int. J. Mol. Sci..

[55] Zheng Y., Li L., Liu Q., Zhang H., Cao Y., Xian M., Liu H. (2016). High-specificity synthesis of novel monomers by remodeled alcohol hydroxylase. BMC Biotechnol..

[56] Chen Z.F., Chen J., Ma N.N., Zhou H.F., Cong Z.Q. (2018). Selective hydroxylation of naphthalene using the H_2_O_2_-dependent engineered P450BM3 driven by dual-functional small molecules. J. Porphyr. Phthalocyanines.

[57] Morris G.M., Goodsell D.S., Halliday R.S., Huey R., Hart W.E., Belew R.K., Olson A.J. (1998). Automated Docking Using a Lamarckian Genetic Algorithm and Empirical Binding Free Energy Function. J. Comput. Chem..

[58] (2010). The PyMOL Molecular Graphics System.

