# Supplementary material for: Construction of Biocatalysts Using the P450 Scaffold for the Synthesis of Indigo from Indole
Source: Int J Mol Sci. 2023 Jan 25;24(3):2395. doi: 10.3390/ijms24032395 (PMC9917246; doi:10.3390/ijms24032395)
Supplement: Supplementary file 1 [file ijms-24-02395-s001.zip › ijms-2143335-supplementary.pdf]

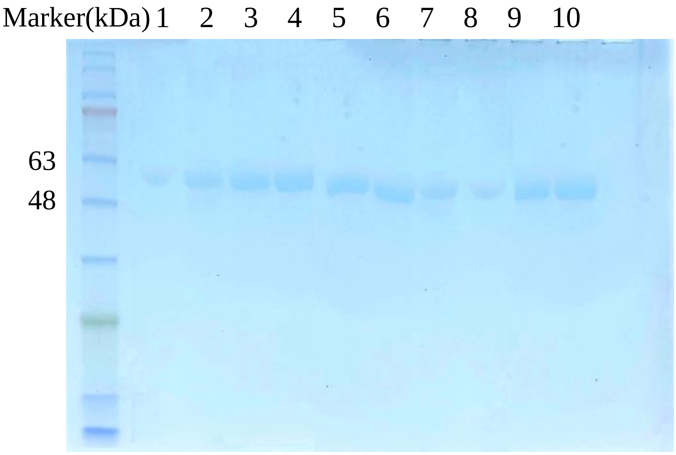

Figure S1. SDS-PAGE of P450BM3 and its mutants. Lane 1-10: F87A, V78A/F87A, F87A/T268A, F87A/T268I, F87A/T268V, F87G, V78A/F87G, F87G/T268A, F87G/T268I, F87G/T268V.

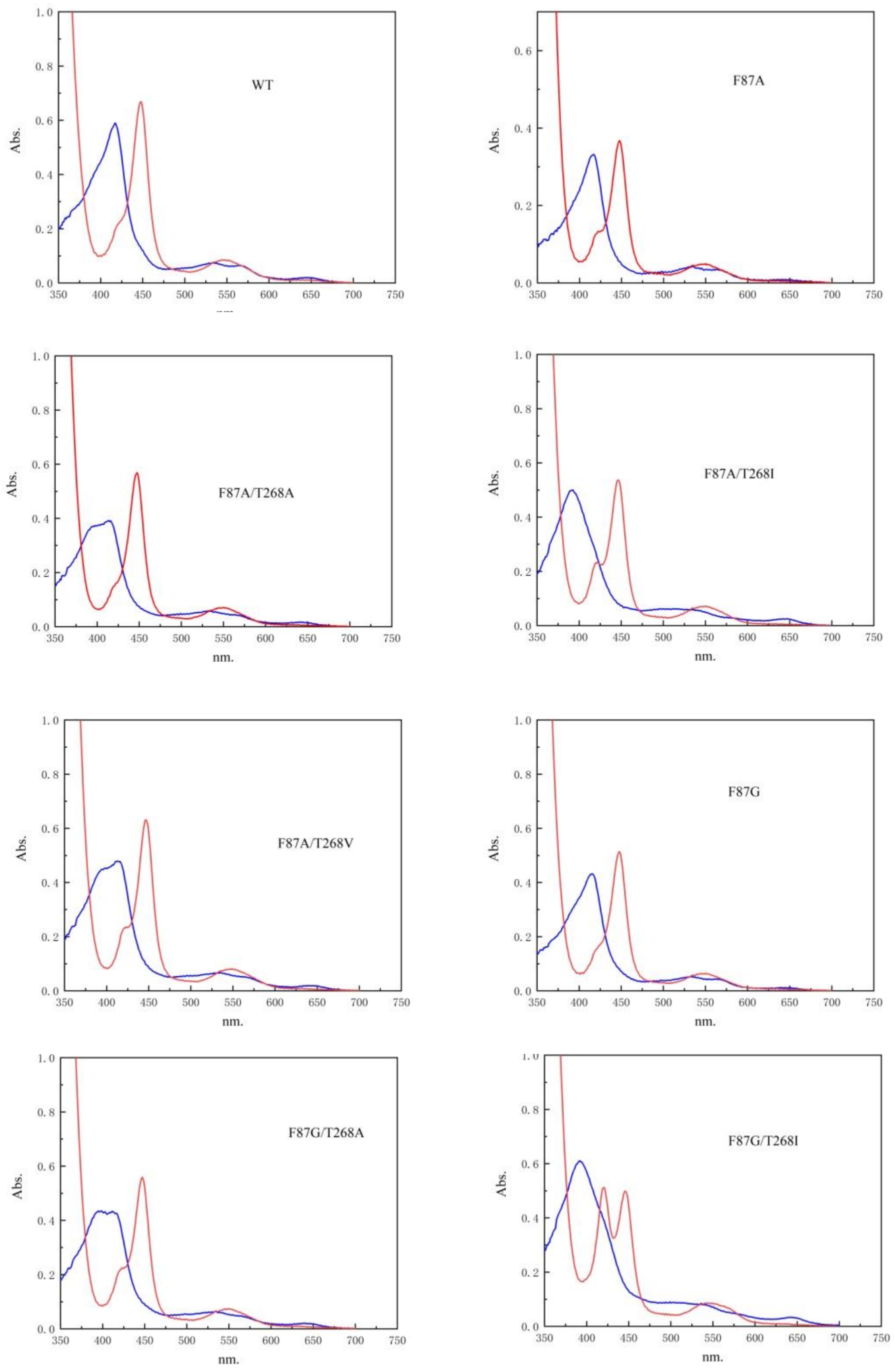

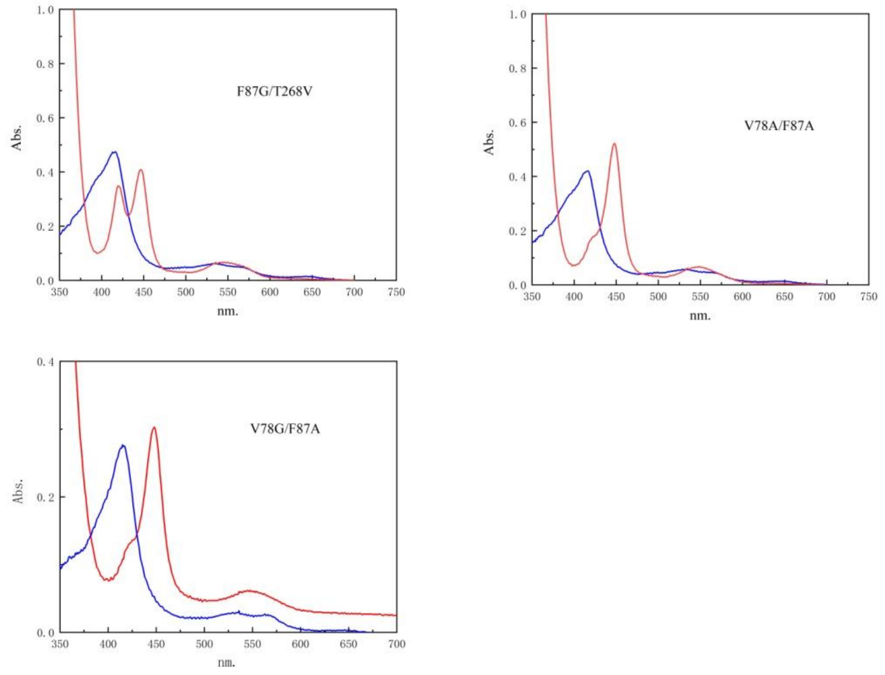

Figure S2. UV-visible spectral changes of the wild type P450BM3 and its mutants (blue line) upon addition of $Na_2S_2O_4$ (red line) for the formation of a ferrous CO complex through the reduction of ferric heme.

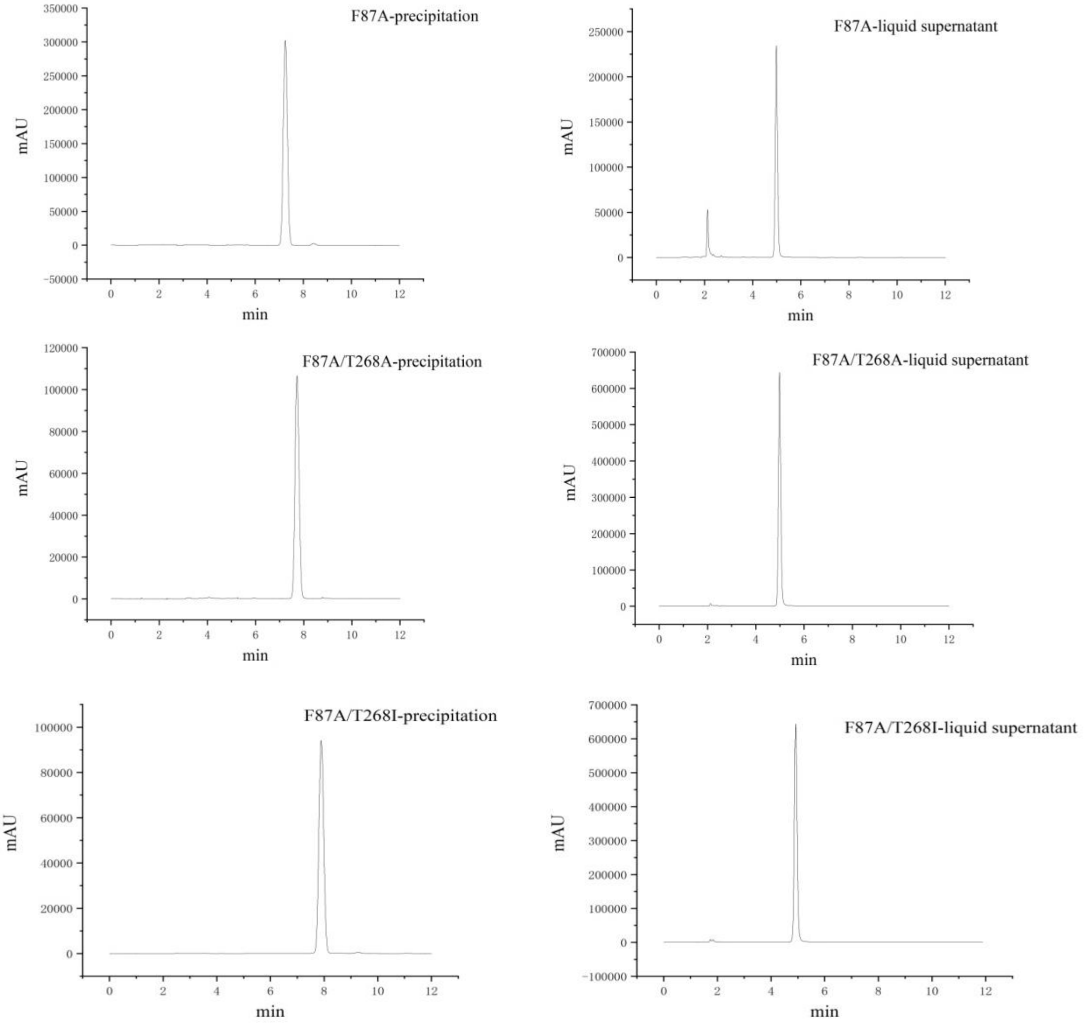

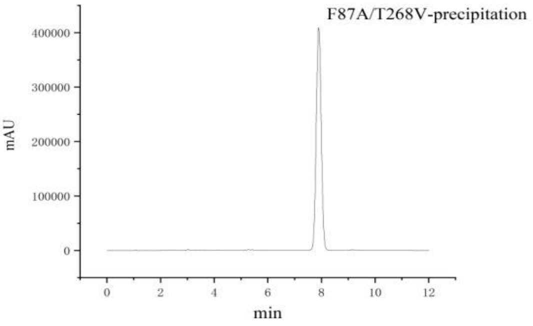
F87A/T268V-precipitation
mAU
min

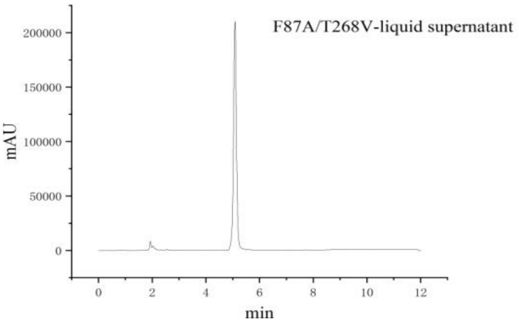
F87A/T268V-liquid supernatant
mAU
min

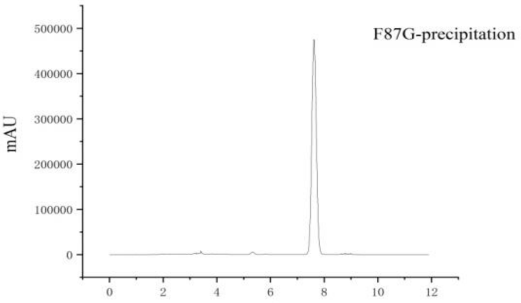
F87G-precipitation
mAU
min

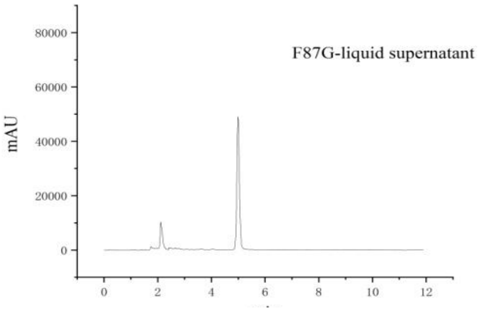
F87G-liquid supernatant
mAU

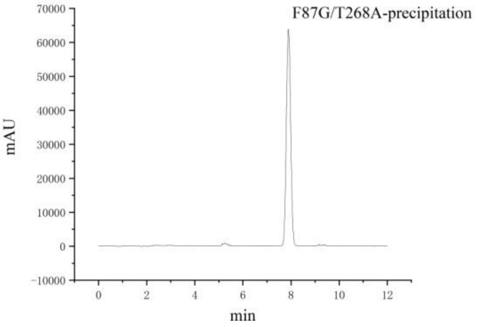
F87G/T268A-precipitation
mAU
min

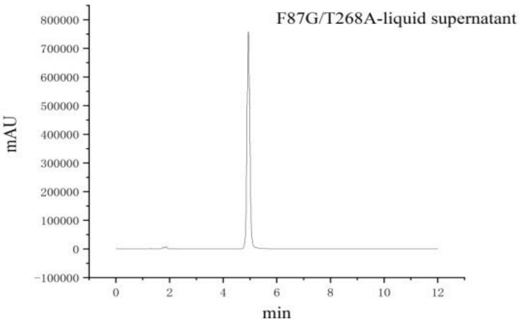
F87G/T268A-liquid supernatant
mAU
min

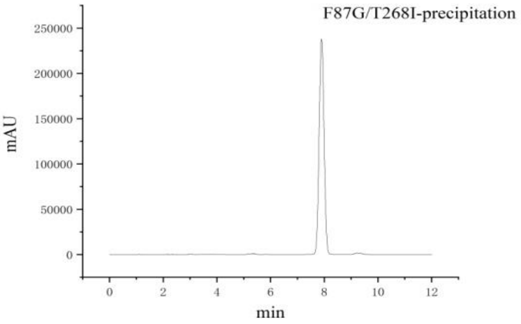
F87G/T268I-precipitation
mAU
min

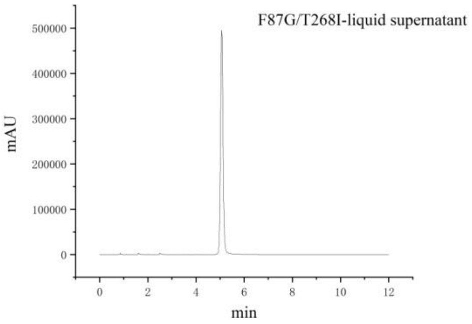
F87G/T268I-liquid supernatant
mAU
min

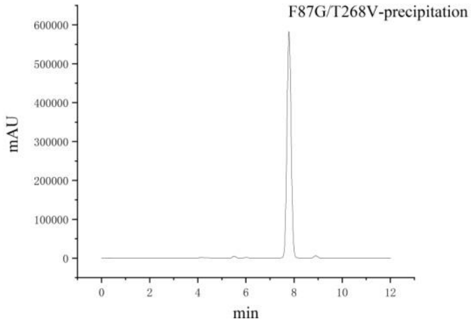
F87G/T268V-precipitation
mAU
min

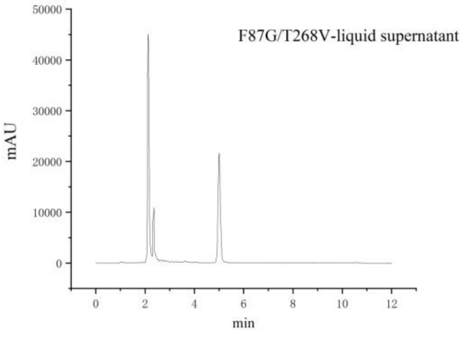
F87G/T268V-liquid supernatant
mAU
min

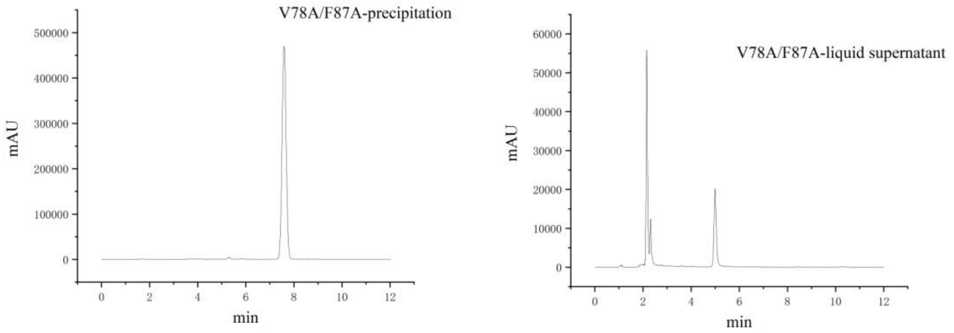

Figure S3. HPLC analyses for the oxidation of indole by P450BM3 mutants in the absence of DFSM. The HPLC analysis of the extract of the reaction mixture monitored at 280 nm gave a couple of peaks assignable to isatin (2.29 min) and oxindole (2.19 min), a peak at 5.00 min corresponding to indole and a peak at 7.91 min corresponding to indigo.

Table S1. Docking energy of indole binding to F87A/T268V P450BM3 mutant.

Model	$E_{binding}$[a]	$E_{inter-mol}$[b]	E_{vdw}[c]	E_{elec}[d]
1	-2.74	-2.74	-2.74	0.00
2	-2.74	-2.74	-2.74	0.00
3	-2.74	-2.74	-2.74	0.00
4	-2.74	-2.74	-2.74	0.00
5	-2.73	-2.73	-2.73	0.00
6	-2.73	-2.73	-2.74	0.00
7	-2.73	-2.73	-2.73	0.00
8	-2.73	-2.73	-2.73	0.00
9	-2.73	-2.73	-2.73	0.00
10	-2.73	-2.73	-2.73	0.00

[a] Binding energy. [b] Intermolecular energy. [c] van der Waals energies. [d] Electrostatic interactions.

Table S2. Docking energy of indole binding to F87G/T268A P450BM3 mutant.

Model	$E_{binding}$[a]	$E_{inter-mol}$[b]	E_{vdw}[c]	E_{elec}[d]
1	-3.88	-3.88	-3.8	-0.08
2	-3.88	-3.88	-3.79	-0.08
3	-3.88	-3.88	-3.8	-0.08
4	-3.88	-3.88	-3.8	-0.08
5	-3.88	-3.88	-3.8	-0.08
6	-3.88	-3.88	-3.8	-0.08
7	-3.88	-3.88	-3.8	-0.08
8	-3.88	-3.88	-3.8	-0.08
9	-3.88	-3.88	-3.8	-0.08
10	-3.88	-3.88	-3.8	-0.08

[a] Binding energy. [b] Intermolecular energy. [c] van der Waals energies. [d] Electrostatic interactions.

Table S3. Docking energy of indole binding to F87G/T268V P450BM3 mutant.

Model	$E_{binding}$[a]	$E_{inter-mol}$[b]	E_{vdw}[c]	E_{elec}[d]
1	-2.49	-2.49	-2.48	-0.01
2	-2.48	-2.48	-2.48	0.00
3	-2.48	-2.48	-2.47	-0.01
4	-2.48	-2.48	-2.48	0.00
5	-2.47	-2.47	-2.48	0.01
6	-2.47	-2.47	-2.48	0.01
7	-2.47	-2.47	-2.47	0.00
8	-2.47	-2.47	-2.47	0.00
9	-2.47	-2.47	-2.46	-0.01
10	-2.46	-2.46	-2.45	-0.02

[a] Binding energy. [b] Intermolecular energy. [c] van der Waals energies. [d] Electrostatic interactions.
